# Strategies to improve safety profile of AAV vectors

**DOI:** 10.3389/fmmed.2022.1054069

**Published:** 2022-11-01

**Authors:** Tuisku Suoranta, Nihay Laham-Karam, Seppo Ylä-Herttuala

**Affiliations:** ^1^ A. I. Virtanen Institute for Molecular Sciences, University of Eastern Finland, Kuopio, Finland; ^2^ Heart Center, Kuopio University Hospital, Kuopio, Finland; ^3^ Gene Therapy Unit, Kuopio University Hospital, Kuopio, Finland

**Keywords:** AAV, gene therapy, capsid engineering, genome engineering, immunogenicity, genotoxicity, adeno-associated viral vectors

## Abstract

Adeno-associated virus (AAV) vectors are currently used in four approved gene therapies for Leber congenital amaurosis (Luxturna), spinal muscular atrophy (Zolgensma), aromatic L-amino acid decarboxylase deficiency (Upstaza) and Haemophilia A (Roctavian), with several more therapies being investigated in clinical trials. AAV gene therapy has long been considered extremely safe both in the context of immunotoxicity and genotoxicity, but recent tragic deaths in the clinical trials for X-linked myotubular myopathy and Duchenne’s muscular dystrophy, together with increasing reports of potential hepatic oncogenicity in animal models have prompted re-evaluation of how much trust we can place on the safety of AAV gene therapy, especially at high doses. In this review we cover genome and capsid engineering strategies that can be used to improve safety of the next generation AAV vectors both in the context of immunogenicity and genotoxicity and discuss the gaps that need filling in our current knowledge about AAV vectors.

## Introduction

The last 10 years have been full of great successes for gene therapy and a lot of this success can be attributed to a small virus named Adeno-associated virus (AAV). It was the delivery method of choice for Glybera, the first gene therapy approved by the European Medicines Agency (EMA) in 2012 (Ylä-Herttuala, 2012). Since then, two more *in vivo* AAV gene therapies have been approved by EMA and the United States Food and Drug Administration (FDA): Luxturna and Zolgensma (Keeler and Flotte, 2019), with Upstaza for aromatic L-amino acid decarboxylase deficiency and Roctavian for Haemophilia A having also secured marketing authorisations from EMA (BioMarin Investors, 2022; PTC Therapeutics, 2022).

**FIGURE 1:**
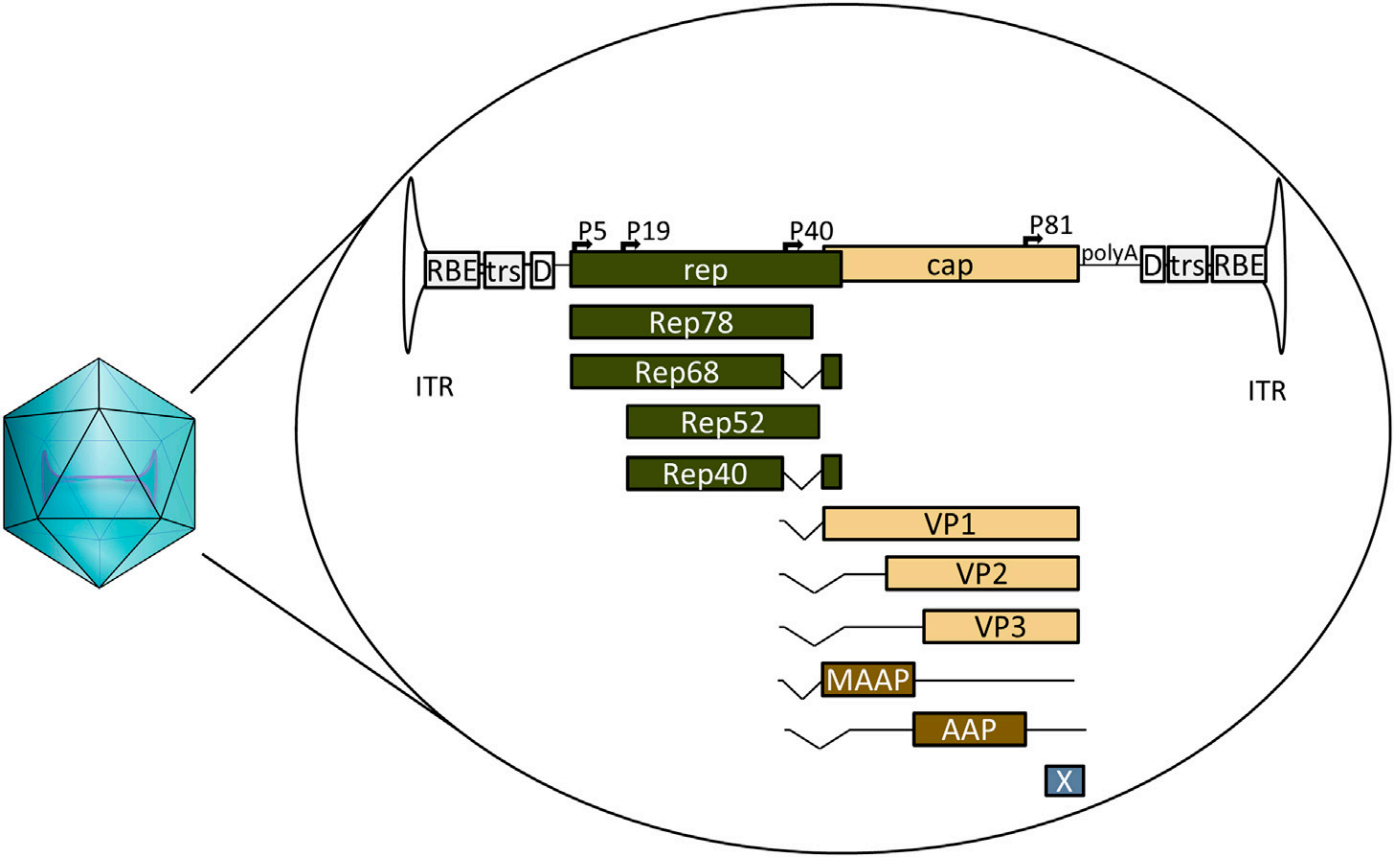
Schematic representation of wild-type AAV2, its encapsulated genome and the known open reading frames. ITR: inverted terminal repeat. RBE: Rep binding element, trs: terminal resolution site D: D-sequence, P5, P19, P40, and P81: viral promoters, Rep78, Rep68, Rep52, and Rep40: proteins coded by rep, involved in genome replication and packaging. VP1, VP2, and VP3: structural capsid proteins coded by cap, MAAP: membrane associated accessory protein, AAP: assembly activating protein, X: protein X, polyA: viral polyadenylation signal.

AAV is a defective parvovirus with a single stranded DNA genome packaged inside a non-enveloped capsid. It is unable to replicate without the presence of a helper virus such as adenovirus or herpes simplex virus (HSV), and instead, in their absence, establishes a latent infection by integrating site specifically into the so-called AAV safe harbour (AAVS1) in 19q13.3 (Atchison et al., 1965; Kotin et al., 1991; Weindler and Heilbronn, 1991). The AAV genome itself contains the bare necessities packed into 4.7 kb (Figure 1). The 145 nt inverted terminal repeats (ITRs) flank both ends of the genome, containing the signals necessary for genome replication and packaging; they are also the only signal required for AAV vectors *in cis*. Between the ITRs reside two genes: the *rep* and the *cap*. For AAV serotype 2 (AAV2) the *rep* codes for four different replicase proteins: Rep78/68, which are responsible for genome replication, and Rep52/40, responsible for genome packaging (King et al., 2001; Stracker et al., 2004). The *cap* codes for three structural proteins: VP1, VP2, and VP3, which form the 22 nm diameter AAV capsid in a 1:1:10 ratio. Three additional proteins have been identified within the AAV2 *cap:* assembly activating protein (AAP), membrane associated accessory protein (MAAP) and protein X (Sonntag et al., 2010; Cao et al., 2014; Ogden et al., 2019; Galibert et al., 2021). The basic structure is the same for all known serotypes, though some differences exist; for example, AAV5 *rep* does not code for Rep68 at all (Fasina and Pintel, 2013).

The natural AAV infection is not associated with any confirmed pathology and is most often established in the liver and bone marrow (Gao et al., 2004). However, the many different serotypes discovered in humans and non-human primates (NHPs) have differing receptor usage, and thus target organs and cell types at varying efficiencies (Wu et al., 2006; Huang et al., 2014). This has been utilised as an advantage in gene therapy since a specific serotype can be selected to optimise transduction of different target tissues. Other attributes also vary–for example AAV9 can cross the blood-brain barrier, while AAV2 cannot (Liu et al., 2021). Conveniently, AAV is also capable of process called cross-packaging, which allows use of AAV2 ITRs in the vector genome, together with AAV2 *rep* provided *in trans,* to be packaged into the capsid of other serotypes, making switching between capsids relatively effortless (Rabinowitz et al., 2002).

AAV vectors possess many attributes that have made it the delivery vector of choice for *in vivo* gene therapy. In addition to the variety of attributes dictated by the naturally occurring serotypes, the repertoire has been further expanded by engineered capsid variants (Büning et al., 2015). The safety profile also appeared excellent based on the animal studies and early clinical trials. The immunogenicity was found to be relatively low, and the risk of genotoxicity minimal, as the gutted vector showed no active integration (Salmon et al., 2014). Yet, despite the lack of integration, the expression is not transient, as the genomes can persist in an episomal form and continue to produce the transgene even after 10 years (Buchlis et al., 2012).

With more trials now conducted and the demand soaring it has become clear that AAV gene therapy also faces challenges. Firstly, the manufacturing capacity that was sufficient for ultra-rare orphan diseases could not support the pipeline for more common disorders. This has meant moving away from production in monocultures to bioreactors and from ultracentrifugation-based purification to high-throughput affinity chromatography. Though these technologies have quickly evolved, the race to meet the increasing industry demands is ever ongoing (Dobrowsky et al., 2021). The analytical requirements too are far from simple–the differences to small molecules and simpler biological products means that the regulatory demands have evolved in parallel with the therapies, yet many processes remain unstandardised (Ramsey et al., 2021). Even determining the dose that the patient is receiving can be tricky, as the measured titres can vary up to ten-fold between different laboratories (Lock et al., 2010; Ayuso et al., 2014).

Although some of the technical challenges have been resolved, alarming new concerns around safety have emerged from the exponentially increasing number of studies and trials. Death of four patients in the Astellas-Audentes trial for X-linked myotubular myopathy (XLMTM), one in Pfizer’s trial for Duchenne’s Muscular Dystrophy (DMD) and the death of one patient administrated with Zolgensma has called into question the high doses used and emphasised the need for AAV vectors with a better efficacy at lower doses. Additional concerns around acute toxicity have arisen from animal studies, with neuropathology and toxicity reported in non-human primates (NHPs) and piglets treated with a high dose of AAV (Hinderer et al., 2018). Furthermore, lesions in dorsal root ganglia were found in the majority of AAV dosed NHPs (Hordeaux et al., 2020). Long term safety profile has also come under scrutiny due to the reports of hepatocellular carcinoma in mice, bile-duct proliferation in rabbits and clonal expansion of transduced hepatocytes in a canine model of Haemophilia A (e.g., Donsante et al., 2001; Bell et al., 2006; Hytönen et al., 2019; Dalwadi et al., 2021b; Li et al., 2021; Nguyen et al., 2021; reviewed in Sabatino et al., 2022). Furthermore, a recent outbreak of hepatitis in Scotland was linked to a wild type AAV2 infection, though the exact connection to the pathophysiology observed remains unclear (Ho et al., 2022).

Safety is of paramount importance for any therapeutic intervention, and the AAV gene therapy field must strive to find–and implement–solutions that address the questions being asked as fully as possible. Here, we have reviewed genome and capsid engineering strategies that can be used to improve the safety profile by promoting immune-evasion, avoiding oncogene activation, and increasing on-target delivery.

## Engineering AAV vectors for better safety profile

Many approaches have been taken to engineer AAV vectors. At the vector genome level, this means adding, mutating and deleting sequences; for example, the self-complementary AAV (scAAV) vectors were designed by deleting key signals from the second ITR, causing the genome replication to continue to copy also the second strand instead of termination (McCarty et al., 2003). Consequently, scAAV vectors are no longer dependent on the second strand synthesis, which is a major rate limiting step in the AAV transduction pathway (Ferrari et al., 1996). Other well-known strategies include codon optimisation of the transgene and the choice and manipulation of elements such as the promoter or polyA sequence.

**FIGURE 2:**
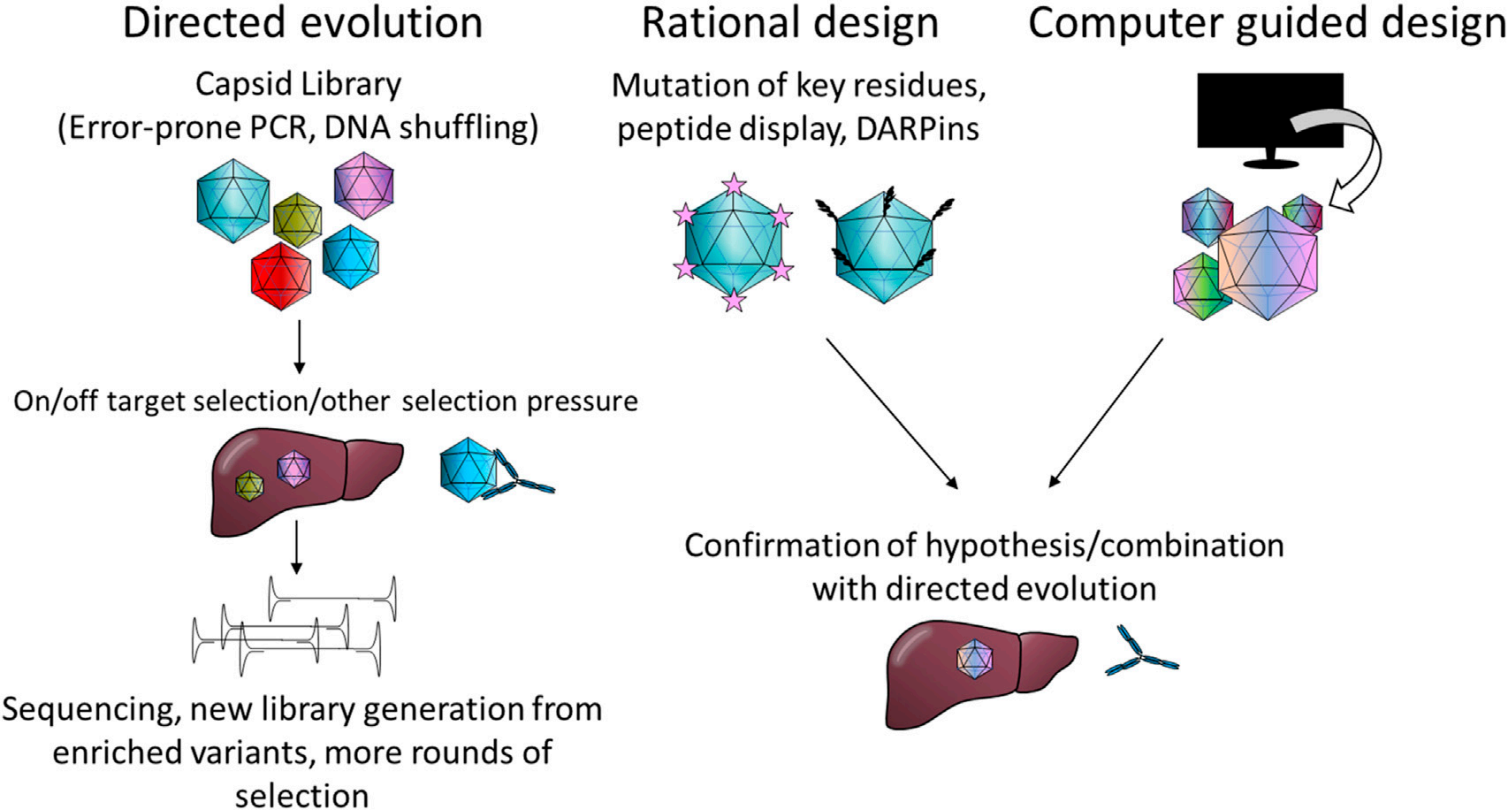
Summary of the most common capsid engineering strategies. PCR; polymerase chain reaction, DARPin: designed ankyrin repeat protein.

Likewise, different strategies have been used for capsid engineering. These can be loosely divided into four categories: directed evolution, rational design, computer guided design and combinations thereof. Commonly, error-prone PCR is used to generate capsid mutant libraries or peptides with known or speculated affinities are inserted into the capsid. The intricacies of these methods are summarised in Figure 2 and reviewed elsewhere in more detail (Buchholz et al., 2015; Li and Samulski, 2020; Zolotukhin and Vandenberghe, 2022).

### Circumventing innate and adaptive immunity

Though AAVs have traditionally been regarded as having low immunogenicity from the safety point of view, we know that this is not the whole story. Two out of the three severe adverse events reported earlier in the Pfizer DMD trial and the patient who died after Zolgensma administration showed signs of atypical haemolytic uremic syndrome (aHUS)-like complement activation, highlighting that the relatively low immunogenicity can regardless become a serious safety concern (Guillou et al., 2022; Pfizer, 2022). Severe innate immunity related toxicity had also been previously reported after a high-dose AAV-PHP.B i.v administration in one NHP (Hordeaux et al., 2018). In contrast, the challenges posed by neutralising antibodies and cytotoxic T cell responses for efficacy have long been acknowledged (Manno et al., 2006; Vandamme et al., 2017; Korpela et al., 2022). However, while for example the lysis of transduced cells by capsid-specific cytotoxic T cells might not be life-threatening when the transduction levels are low, this is likely to be different when the majority of the target organ is transduced. Additional complications arise if an immune response is mounted against the transgene, which not only will reduce the therapeutic efficacy but may also compromise any protein replacement therapy that the patient has previously relied upon.

**FIGURE 3:**
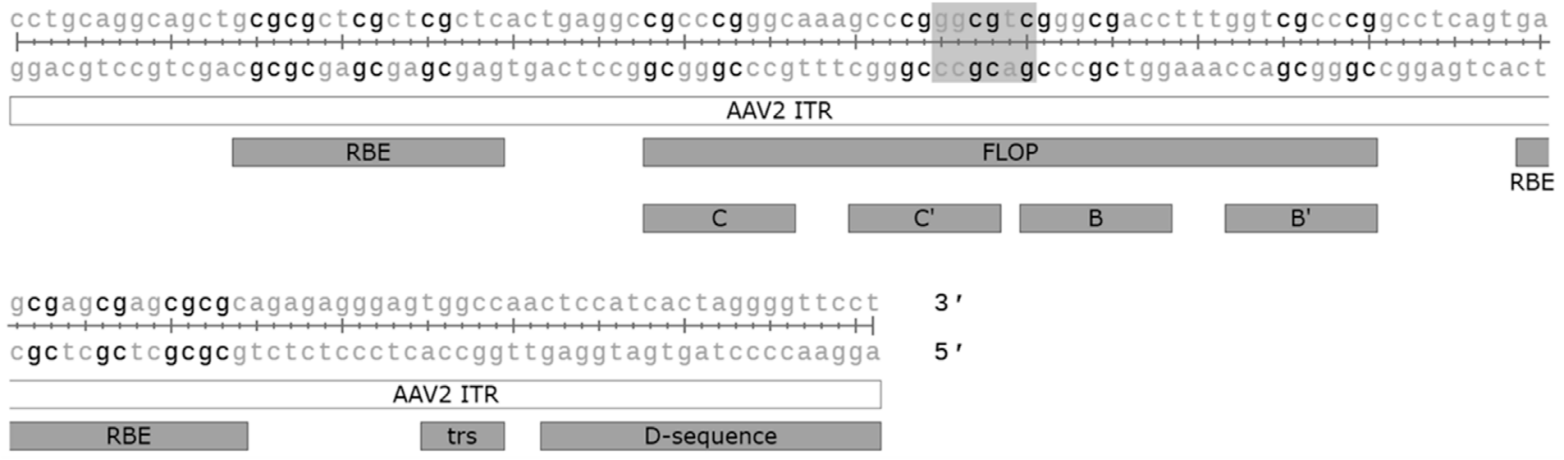
CpG motifs (in black) and the optimal mouse TLR9 recognition motif (highlighted) found in the AAV2 ITR sequence. ITR: inverted terminal repeat. Flop: hairpin sequence orientation, C, C′, B and B′: hairpin elements, RBE: Rep binding element, trs: terminal resolution site. Figure generated with SnapGene.

Unmethylated CpG motifs in microbial DNA belonging to the Pathogen associated molecular pattern (PAMP), can be sensed by the intracellular innate sensor Toll-like receptor 9 (TLR9). Unmethylated CpG DNA has also been linked to complement activation, which seems to be mediated *via* both TLR9 dependent and independent mechanisms (Mangsbo et al., 2009). CpG motifs are often unmethylated in AAV vectors, despite the production in mammalian cells, and the activation of the TLR9-MyD88 signalling pathway by unmethylated CpG has been shown to promote adaptive immune responses not only against the capsid but also the transgene (Zhu et al., 2009). Studies in mice have shown that modifying the transgene sequence to eliminate CpG can lead to increased transgene expression and persistence, likely specifically due to the reduced cytotoxic T-cell responses (Faust et al., 2013; Bertolini et al., 2021). Similar results were also obtained by the incorporation of TLR9 oligonucleotide antagonist sequence directly into the AAV vector genome (Chan et al., 2021). In addition to the transgene, CpG motifs are also present on various other vector elements, such as the widely used cytomegalovirus (CMV) enhancer and promoter, so aiming to minimise the amount of CpG motifs in the whole genome could offer further benefit. The ITRs of AAV2 alone contain 16 CpG motifs each, which may contribute to TLR9-sensing. In their recent study (Pan et al., 2022) successfully modified the ITRs to eliminate all the CpG motifs, though this came at the cost of 3-fold reduced productivity due to reduced genome replication. The effect on immune activation was also not confirmed. Indeed, although TLR9 mediated immunity against the AAV vector genome was hypothesised to have played a part in the loss of expression seen in the BAX 335 Haemophilia B trial (Konkle et al., 2021), the relevance of TLR9 mediated CpG sensing to AAV immunogenicity in humans is not yet clear. There are known differences between human and mouse TLR9 sensing that should be considered: the expression pattern of TLR9 is much more restricted in humans, and the optimal recognition motif in humans (5′-TCGTW-3′) is markedly different from that of mice (5′-RRCGYY-3′) (Huang and Yang, 2010). The AAV2 ITRs, for example, contain one optimal TLR9 recognition motif for mice, but none for humans (Figure 3). Different formulas for estimating the risk of TLR9 activation by vector genome sequences have been proposed, with retrospective analysis of clinical trial data supporting their usefulness (Wright, 2020). Two other factors also contribute to TLR9 sensing: DNA structure and the dose. It is known that self-complementary AAV vectors are recognised by TLR9 more strongly than their single stranded counterparts, but due to their improved efficacy scAAVs may also allow for the use of a lower dosage, potentially compensating for this difference (Martino et al., 2011).

TLR9 is not the only innate immunity sensor recognising AAV vectors–the dsRNA sensor MDA5 has also been implicated (Shao et al., 2018). This seems to be mediated by antisense transcriptional activity from the sequences within and near the ITRs; one study mapped transcriptional initiation at nucleotides 109 to 145 of the ITR while another identified a binding site for the transcription factor HNF1-α just beyond the D-sequence (Haberman et al., 2000; Logan et al., 2017). As the HNF1-α binding site is outside the actual ITRs it can be safely removed, but it is currently present on some vector plasmids, such as the traditional pSub201. The initiation from within the ITR is trickier as the implicated region contains many important elements, such as the Rep binding element (RBE), terminal resolution site (trs), and the D-sequence, required for packaging (Figure 3). However, adding a polyA element facing away from the 3′-ITR, at the opposite orientation to the transgene, can be used to halt any antisense transcription (Shao et al., 2018), though at the cost of space in the vector.

Many of the strategies tested to avoid immune activation have centred on capsid modifications. For now, activation of a third innate immunity sensor, TLR2, which recognises AAV capsid, is not well enough understood to be circumvented by engineering approaches. Instead, several strategies focus on avoiding the adaptive immune responses. Crucially, adaptive T-cell responses rely on antigen presentation by Major Histocompatibility Complex Class I and II molecules, and several of the major epitopes on AAV1, AAV2, and AAV8 have been mapped (Hui et al., 2015), enabling rational design approaches to modify these sequences. Prediction tools are available for both MHC Class I and II epitopes (Nielsen et al., 2010; Paul et al., 2020)—though the latter are more variable and thus harder to predict–and can be used to screen not only other naturally occurring serotypes, but can also be used in designing engineered novel variants.

MHC presentation can also be avoided *via* a second strategy: by circumventing AAV degradation in the proteasomal pathway, which leads to the generation of peptide-epitopes that can be loaded for antigen presentation. Phosphorylation of certain residues acts as a signal for ubiquitination and proteasomal targeting, so mutation studies have been carried out on the AAV2 capsid surface exposed tyrosine (Y), serine (S) and threonine (T) residues, which can be phosphorylated by different kinases (Zhong et al., 2008; Aslanidi et al., 2012; Aslanidi et al., 2013; Li et al., 2015). From the various mutants screened Y444 + 500 + 730F + T491V showed best transduction (Aslanidi et al., 2013), and though this mutant specifically was not studied in the context of T cell activation, the Y444 + 500 + 730F triple mutant transduction was confirmed to result in less MHC Class I presentation than transduction with the wild type (Martino et al., 2013). Additionally, mutations of the surface lysine (K) residues, which undergo ubiquitination, have been studied, with AAV2 K556E performing the best *in vitro* and *in vivo* hepatic gene transfer (Li et al., 2015). Interestingly this study also found that mutating the same lysine residues in AAV8 did not result in similar improvement *in vivo* as was seen for AAV2. Further studies have identified residues on AAV1, AAV3, AAV5, AAV6 and AAV9 capsids that can be mutated to similar effects, even if direct cross-application of specific mutations between the serotypes is not always possible (Cheng et al., 2012; Martino et al., 2013; Sen et al., 2013; Song et al., 2013).

Due to the adverse effects observed in the clinic we now know that complement activation may pose a serious risk to the patients (Guillou et al., 2022; Pfizer, 2022; Solid Biosciences, 2022). The complement system can be activated *via* three different pathways: classical, lectin mediated and alternative, which all ultimately promote target opsonisation, phagocytosis and increased inflammation (Sarma and Ward, 2011). Blood work from patients with adverse effects in response to AAV9 gene therapy has specifically implicated alternative pathway activation (Guillou et al., 2022; Pfizer, 2022). The alternative pathway is activated by hydrolysis of complement factor C3 in the absence of factor H (or other co-factors) interactions with factor I (Meri, 2016). AAV2 has been shown to activate the complement system, and to interact with both factor H and C3, which together with evidence of catabolism of C3b led to the conclusion that the activation happened primarily *via* the classical pathway (Zaiss et al., 2008). Interestingly, another co-immunoprecipitation study with AAV6 found no specific complement binding (Denard et al., 2013), suggesting that once again the differences between the different serotypes might be significant. As the clinical data so far has specifically implicated AAV9 in the alternative pathway activation, there is need to better understand the differences in complement activation between the serotypes and to also apply this understanding to capsid engineering approaches.

In the classical pathway the target is recognised by the binding of IgM or IgG antibodies, with their Fc regions interacting with the components of the complement system, leading to its activation, and boosting both humoral and cellular adaptive immune responses (Sarma and Ward, 2011). Antibodies also directly interact with different immune cells, promoting inflammation, or block transduction by preventing interactions between the virions and the host cell (Klasse and Sattentau, 2002). Clinical trials have so far been mainly focused on screening for these neutralising antibodies (NAbs) due to the concerns around the loss of efficacy, but while considering the safety it is important to remember that also non-neutralising antibodies interact with the immune system. It is generally difficult to avoid existing anti-capsid antibodies simply by switching a serotype, as the antibody epitopes are fairly well conserved between the different serotypes, resulting in high cross-reactivity (Boutin et al., 2010). Several studies have utilised directed evolution approaches combined with libraries generated by error-prone PCR, DNA recombination (e.g., capsid-shuffling) or random peptide ligation to generate variants that can escape NAbs (e.g., Maheshri et al., 2006; Perabo et al., 2006; Bartel et al., 2012). In these approaches the evolutionary pressure (NAbs) is added either *in vitro* or *in vivo* and the capsids that are able to efficiently escape and mediate transduction are chosen for further screening. As this method requires large amounts of screening and some luck in generating the mutants, the more precise rational approaches have also been employed. Based on 3D modelling Girod *et al.,* 1999 generated AAV2 peptide insertion mutants and screened them against different AAV antibodies: A20, C37-B, D3 and C24-B. Peptide insertions at AAV2 residues 261, 381, 534, 573, and 587 were found to decrease the binding of the different antibodies. In further testing by Huttner *et al.,* 2003 AAV2 vectors with peptide insertions in 534, 573, and 587 were able to transduce different cell lines in the presence of antibody containing human sera, while also demonstrating new tropisms based on the inserted peptide. More precise mapping of the immunogenic epitopes has been achieved by cryo-electron microscopy 3D image reconstruction (Gurda et al., 2012; Gurda et al., 2013; Emmanuel et al., 2022). Key proof of concept study was carried out with AAV8. The footprint of AAV8 NAb ADK8 was mapped to capsid residues 588–592, with mutations in this region allowing evasion of ADK8 neutralisation (Gurda et al., 2012). Furthermore, it was demonstrated that after screening of peptide insertion library at the position 590 the tropism for mouse liver was further enhanced (Raupp et al., 2012).

### Minimising the risk of genotoxicity

While the wild-type AAV integrates into the genome in a site-specific manner, this activity is mediated by the large Rep proteins (Stracker et al., 2004), the sequence of which are not present in the gutted vector genomes. Thus, unlike retroviruses, recombinant AAV does not actively integrate, which led many to consider the risk of genotoxicity negligible. Although there is currently no evidence of genotoxicity from any human clinical trials, the observations of hepatocellular carcinoma in mice and clonal hepatocyte expansion in dogs have advocated for a more cautious approach (e.g Donsante et al., 2001; Bell et al., 2006; Dalwadi et al., 2021b; Li et al., 2021; Nguyen et al., 2021). The random integration has been estimated to happen at frequency of 0.001–3%—a fairly rare event by the average estimate (Nowrouzi et al., 2012; Dalwadi et al., 2021a). However, if a 10 kg patient is dosed with 1 × 10^14^ vg/kg, this would mean 1 × 10^10^ integration events even by the most conservative value. The integration logically happens mostly in the areas of open chromatin and seems to be mediated by interactions between the naturally occurring random DNA breaks in the host genome and the recruitment of host DNA repair factors to the AAV genome (Miller et al., 2004). Slightly different DNA repair proteins have been shown to interact with ssAAV and scAAV vectors (Cataldi & McCarty, 2010), however we currently do not know whether one might offer a lower genotoxicity risk than the other. Genome integrations by both ssAAV and scAAV vectors have been implicated in different studies (e.g., Donsante et al., 2007; Rosas et al., 2012), even if no direct comparisons of safety have been carried out thus far.

The major genotoxicity risk events can be roughly divided into two categories: silencing of tumour suppressor genes and oncogene activation. These can be countered by either fully avoiding integration or by controlling it, the latter being easier to achieve. In their 2012 study (Wang et al., 2012) added flanking rDNA homology arms to an AAV vector, which then exhibited highly efficient targeted integration and gene correction. Interestingly, the AAV mediated homologous recombination (HR) exhibits significantly higher efficiency than classical HR (Melo et al., 2014). However, the homology arms demand significant space, substantially reducing the already limited packaging capacity. Alternative approaches have been designed to combat this. Barzel et al. (2015) eliminated the promoter requirement by using HR to hitchhike the payload to the end of albumin gene together with a 2A sequence enabling the generation of both albumin and the payload protein from the endogenous albumin promoter. The whole cassette can also be used as a homology template, simply containing the correction(s) required in the parent gene, as was demonstrated in the correction of epidermolysis bullosa causing point mutation in the LAMA3 gene in keratinocytes (Melo et al., 2014). Combinational strategies with CRISPR-Cas9 have also been used for example *in vitro* stem cell editing and also *in vivo* (Dever et al., 2016; Yang et al., 2016)*.* These strategies are somewhat limited in their applicability and reliance on HR, and the utilisation of the natural integration by wtAAV into the AAVS1 seems like an attractive possibility to explore. However, this requires the presence of Rep78/68, which is difficult to fit into an AAV vector. To solve this both AAV/adenovirus and AAV/HSV hybrids have been investigated (Recchia et al., 1999; Heister et al., 2002), but as they only carry parts of the AAV genome packaged into a different viral vector, they can hardly be classified as AAVs.

**FIGURE 4:**
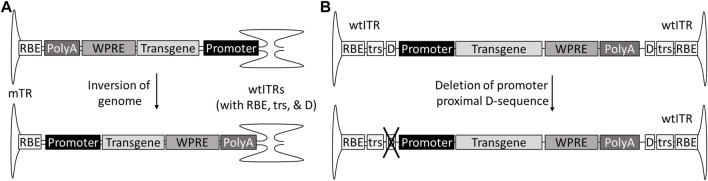
Genome designs to prevent insertions of partial genomes without transcriptional termination signal. The risk of insertional mutagenesis can be reduced by inverting the genome of self-complementary AAVs with promoter to the mTR **(A)** or deleting the promoter-proximal D-sequence from single-stranded AAVs **(B)**.

A part of the genotoxicity risk has been attributed to partial genomes, containing the enhancer-promoter elements without the transcription termination signals (Zhang et al., 2021). These partial genomes can be due to so-called snap back genomes (SBGs) formed during replication when the genome loops back on itself prematurely, forming a self-complementary vector with only part of the sequence. This in turn can be caused for example by hairpin structures such as shRNAs (Xie et al., 2017). Single stranded genomes truncated at the 5′-end are also fairly common, especially in oversized constructs, as the packaging is initiated at the 3′-end ITR (Wu et al.,2010; Tran et al., 2020). For scAAVs, the generation of promoter-only SBGs can be avoided by inverting the genome so that the promoter is proximal to the mutated terminal repeat (mTR), which cannot initiate the packaging (Zhang et al., 2021). In the case of ssAAVs the solution is not as simple; AAV packages genomes of both polarities, meaning both ends of the genome can be truncated. However, this could theoretically be solved by mutating one of the D-sequences required for packaging initiation, and, similar to scAAV, placing the promoter next to the mutated ITR and transcription termination signal, such as polyA, towards the wtITR (Figure 4). As an additional benefit, the deletion of the D(+)-sequence eliminates the binding of NF-κB-repressing factor (NRF), which normally inhibits viral transgene expression (Ling et al., 2015). However, due to half of the genomes present not being available for packaging, this does come at the expense of productivity.

The choice of specific elements in the vector can also either reduce or enhance the risk of genotoxicity. Certain promoters, for example, seem to have a higher risk of genotoxicity than others. In a study of hepatocellular carcinoma in mice, chicken beta actin (CBA) and thyroxine-binding globulin (TBG) enhancer/promoter elements were associated with HCC formation, while human α-1 antitrypsin (hAAT) promoter was not. This was speculated to be due to the increased activation of nearby genes by CBA and TBG promoters (Chandler et al., 2015). It is also known that many enhancers and promoters possess bi-directional activity (Wei et al., 2011). Insulators have been used in retro- and lentiviral vectors to address this and also to minimise read-through transcription, which is another concern for integrated AAV genomes (Browning and Trobridge, 2016). A study by Fitzsimons *et al.,* 2001 also tested insulators in the context of AAV, designing a doxycycline controlled, insulator flanked cassette, which was found functional in rat brain. Indeed, such inducible promoters may also be suitable for improving safety for some therapeutic applications (Chen et al., 2013).

A commonly included element in the AAV vector genome is woodchuck hepatitis virus post-transcriptional regulatory element (WPRE), which promotes transgene expression by supporting transcriptional termination and enhancing mRNA nuclear export (Loeb et al., 1999; Higashimoto et al., 2007). The wild-type WPRE does, however, come with concerns of oncogenesis, as it contains in its beta-element verified promoter activity and the start of the WHV protein X (WHX) ORF. Truncated WHX fragments have been linked to oncogenicity in liver tumours (Wei et al., 1995; Tu et al., 2001). The WHX We1 promoter/enhancer element contained within the wt WPRE sequence has also been validated to be active in the context of vectors (Wei et al., 1995; Kingsman et al., 2005). To avoid this, mutants have been generated with comparable activity, including the so-called mutant 6, with both the promoter and start codon mutated (Zanta-Boussif et al., 2009). A shorter alternative also exists in the so-called mutant 3, which completely lacks the beta-element, providing a safer and space saving alternative (Choi et al., 2014). The use of these mutated WPRE sequences seems then advisable as the enhanced expression can support the use of smaller doses, and the more efficient transcriptional termination reduces the risk of transcriptional read-through.

### Controlling on-target delivery and off-target expression for better overall safety

Dose affects all aspects of safety; the higher the dose, the higher the chance of immune activation and genome integration. Recent clinical trials utilising very high doses have used systemic delivery to target muscle (Duan*,* 2018; Shieh et al., 2020). In this case direct delivery to the target tissue is not feasible but comes with a widespread off-target transduction and thus loss of efficacy at the target tissue. Notably, systemic delivery means the vector is circulated through the liver, which most serotypes have some tropism for. In many cases this results in the rise of transaminases, markers of liver toxicity, which is one of the most common adverse effects in AAV clinical trials (Colella et al., 2018). However, some liver transduction may also be beneficial, as transgene expression in the liver seems to promote tolerogenicity (LoDuca et al., 2009; Fuchs et al., 2020). To take advantage of this Colella *et al.* (2019) multiplexed tissue specific regulatory elements to create liver-muscle and liver-neuron specific tandem promoters, which successfully prevented anti-transgene immunity in mice. Many engineering strategies have focused on the on-target delivery, but it seems that now the focus needs to shift towards reducing off-target delivery. This means selecting clones that have the best transduction in the target tissue *in vivo* and the least in the control tissues–or similarly screening both the target cell type and any relevant off-targets *in vitro.* It is acknowledged that the translatability of the selection in the *in vitro* cell-based and the *in vivo* animal models to humans can be tricky; hence it is imperative that rational design approaches can also take off-target effects into consideration either at the design stage or later during the screening.

Capsid engineering can come with additional sets of problems in productivity and downstream processing that can hinder translation into the clinics. Alternatively, the expression of the transgene can be limited by using tissue specific promoters or other regulatory elements that induce gene expression in cell-type specific manner (Pacak et al., 2008; Wang et al., 2008; Mushimiyimana et al., 2021). Hence, in systemic delivery of these AAVs despite the high amounts of delivery to non-target tissues, the limited promoter activity can reduce the risk of genotoxicity and toxicity associated with the transgene expression. This has been observed in the context of ocular delivery where the use of non-specific or RPE-specific promoter led to strong RPE toxicity, while the transduction by photoreceptor specific promoter was well tolerated (Xiong et al., 2019).

## Discussion

The decades of study into AAV gene therapy have generated a lot of insight and strategies into how to manipulate the AAV genome and capsid for therapeutic benefit. The tropism of the wild type virus can be engineered to suit our needs better by the tools of directed evolution and rational design, and we can make more informed choices on how to design the vector genome as more details become available. Here, in the light of the recent concerns, we have focused on covering the safety aspect, but it is notable that many of the strategies that improve safety can also improve efficacy. For example, CD8^+^ T-cell responses cause loss of transgene expression, and off-target delivery results in the “waste” of the vector, requiring higher dosage. We have given an overview of the challenges and the potential solutions suggested in Table 1.

**TABLE 1:** Genome and capsid engineering solutions for different safety aspects of AAV gene therapy.

Immunogenicity		Genotoxicity
Innate immunity	Adaptive immunity	
TLR9 sensing	MHC presentation	Random integration
- Reducing vector CpG content (Faust et al., 2013)	- Avoidance of proteasomal targeting by mutating key residues (Cheng et al., 2012; Aslanidi et al., 2013; Martino et al., 2013; Sen et al., 2013; Song et al., 2013)	-Homology directed targeted integration and genome editing (Wang et al., 2012; Melo et al., 2014; Dever et al., 2016)
- Inhibitor oligonucleotide sequences (Chan et al., 2021)	- Mutation of MHC presented peptides (Hui et al., 2015)		
MDA5 sensing		Promoter/enhancer driven oncogene activation	
- Eliminating HNF1-α binding site near the ITR sequence (Logan et al., 2017)	Anti-capsid antibodies		
- Inserting a polyA sequence to block ITR originating antisense transcripts (Shao et al., 2018)	- Elimination of antibody recognition by rational design or by directed evolution (Zaiss et al., 2008; Denard et al., 2013)	- Keeping the genome size within the wt virus size to minimise partial genome packaging (Wu et al., 2010)		
Complement activation	Anti-transgene responses	- Use of tissue specific promoters (Wang et al., 2008; Pacak et al., 2009; Xiong et al., 2019)		
- Alternative pathway: choosing/engineering serotypes which will not trigger alternative pathway (Zaiss et al., 2008; Denard et al., 2013)	- Induction of liver tolerance, e.g. by using a tandem promoter (Colella et al., 2019)	- scAAV: inverting the genome with promoter proximal to the mTR (Zhang et al., 2021)		
- Classical pathway: engineering to eliminate anti-capsid antibody epitopes (Maheshri et al., 2006; Perabo et al., 2006; Bartel et al., 2012)	- Minimising overall vector immunogenicity (Colella et al., 2019)	- ssAAV: D-sequence deletion at promoter proximal ITR (Zhang et al., 2021; Ling et al., 2015)	
		WHV protein X oncogenicity
		- WPRE mutants without the WHVX transcription, (Zanta-Boussif et al., 2009; Choi et al., 2014; Ling et al., 2015; Zhang et al., 2021)		

All: Minimising dose by increasing efficacy and maximising on-target delivery by capsid engineering (Buchholz et al., 2015; Büning and Srivastava, 2019)

The utilisation of many of these strategies does not, of course, come without challenges. In genome engineering the biggest hurdle is often space; the already limited packaging capacity of AAV is usually utilised to the full, with little room for additional elements or large tissue-specific promoters. For capsid engineering the problem is multifactorial: the yield may not be as good as for the natural serotypes, purification with existing affinity chromatography columns not possible, and the regulatory demands higher. In many cases the ideal elements may be patented, requiring additional licensing. Additionally, the increasing complexity places more demands on the expertise required at multiple different fronts.

Naturally, safety is a complex issue that cannot be purely solved by vector design and may even be influenced by factors that we have not even yet considered. For example, recent studies have compared the axonal transport and anterograde transneuronal spread of the serotypes, finding differences that could have important implications for safety and clinical trial design (Aschauer et al., 2013; Zingg et al., 2017; Yu-Wai-Man et al., 2020). Even the immunomodulatory regimens chosen could play a vital role in determining whether the therapy is successful as wrongly chosen regimen can potentially block the induction of tolerance (Samelson-Jones et al., 2020). At the same time many issues are already well documented, such as the number of empty capsids and their influence on immunogenicity (Verdera et al., 2020). While genome and capsid engineering can be used to alleviate these issues, more solutions are still required at the downstream processing stage (Srivastava et al., 2021). In the animal studies genotoxicity has been strongest in neonatal animals or ones with HCC predisposition (Sabatino et al., 2022), and pre-existing hepatobiliary condition seems to have played a role at least in three of the serious adverse effects observed in the ASPIRO trial (Audentes Therapeutics, 2020). As such we need to gain more understanding on what risk factors to consider and screen for in patients. Sex also seems to matter; liver transduction with AAV2 and AAV5 was significantly higher in male mice than in females, whereas female mice had higher AAV9 transduction in the brain (Davidoff et al., 2003; Maguire et al., 2013). How this translates to human patients remains to be seen.

Bringing more of these insights of AAV into the clinical trials should be a top priority together with continuing the research to gain more in-depth understanding of the underlying biology. Currently, little testing has been done to combine any of the multitude of options covered in this review and seeing the feasibility of these in an “optimally safe AAV” would certainly be of interest to many. For now, the “store-bought” standard fare is then the easiest solution on many accords, but we should consider that in the future it may not be enough, especially as gene therapy aims to expand to target more common, less debilitating diseases. If we want to see more AAV gene therapies succeeding these challenges need to be faced head on, as we aim for safer, more efficacious, and cost-effective therapies.
